# A concise synthesis of Fingolimod: an orally available drug for treating multiple sclerosis

**DOI:** 10.1186/s13065-015-0081-8

**Published:** 2015-02-01

**Authors:** Ning Yan, Kai Chen, Xinfa Bai, Lei Bi, Lei Yao

**Affiliations:** School of Pharmacy, Yantai University, Yantai, Shandong 264005 China; School of Chemistry and Chemical Engineering, Anhui University of Technology, Maanshan, Anhui 243002 China

**Keywords:** Fingolimod, 3-nitropropionic acid, Immunosuppressant

## Abstract

A concise route for the synthesis of Fingolimod is reported. Starting from n-octylbenzene and 3-nitropropionic acid, a sequence of reactions consisting of Friedel-Crafts acylation, reduction, and double Henry reaction, followed by hydrogenation were applied to prepare Fingolimod with a yield of 31%, and an overall atom economy of 82.7%.

**Graphical Abstract:**
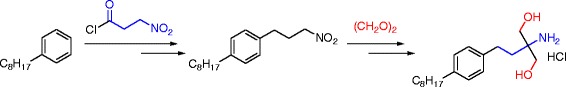
Starting from 3-nitropropanyl chloride, Fingolimod was obtained in 4 steps with an overall yield of 31%.

Starting from 3-nitropropanyl chloride, Fingolimod was obtained in 4 steps with an overall yield of 31%.

## Findings

Fingolimod (1, FTY-720) was first synthesized in 1992 [1,2]. It is an immunomodulating drug, and was approved for treating multiple sclerosis(MS) in 2010. Fingolimod is a sphingosine 1-phosphate receptor modulator precursor that becomes active in vivo following phosphorylation by sphingosine kinase 2 to form Fingolimod-phosphate. This phosphate moiety binds to extracellular G protein-coupled receptors, sphingosine 1-phosphates and prevents the release of lymphocytes from lymphoid tissue thus preventing them from contributing to an autoimmune reaction. This process could lead to a neural protection and restoration process, and can reduce MS recurrence rate, slow down the progression of damage, reduce intracranial magnetic resonance imaging (MRI), the number of lesions, and reduce the severity of the lesions [3,4].

**Figure 1:**
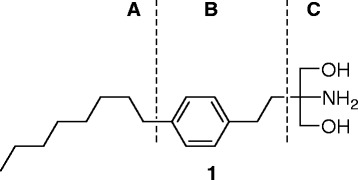
**Structure of Fingolimod.**

Structurally, Fingolimod could be divided into three parts: the hydrophobic *n*-octyl side chain (A), the planar aromatic ring with a two carbon linker (B), and the hydrophilic amino-alcohol terminal (C, Figure 1).

To date, several synthetic routes for Fingolimod have been reported [5-15]. However, these synthetic methods were unsuitable to industrial scale-up mainly due to the involvement of multiple steps and dangerous chemicals such as lithium aluminium hydride (LAH).

**Scheme 1:**
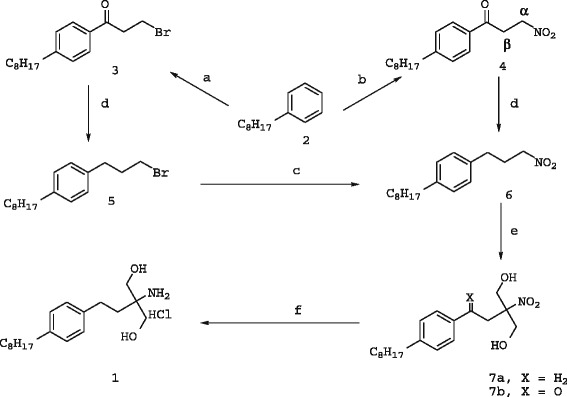
**Synthesis of Fingolimod.**

Discovery of efficient synthetic methods for active pharmaceutical ingredients is always a medicinal chemist’s interest. During our investigation for the application of 3-nitropropionic acid as synthetic building block, we envisioned that compound **4** (Scheme 1) could be a key intermediate in the synthesis of Fingolimod. This compound could be obtained by other synthetic routes reported in literatures [16]. Herein, we report a concise synthesis of Fingolimod, which is economically sound and easy to operate.

Treatment of the commercially available *n*-octylbenzene (**2**) with 3-nitropropanoyl chloride under AlCl_3_ gave compound **4** with good yield (85%). The *gem*-hydroxylmethyl moiety was introduced to compound **4** on the alpha carbon to form intermediate **7b**, and then the nitro group and carbonyl group were reduced simultaneously in one single step. However, the reaction of compound **4** with formaldehyde (or equivalent) was messy, and no clean **7b** could be isolated. We ascribed the failure to the presence of the carbonyl group, which might cyclize with the hydroxyl group generated in the Henry reaction, or form a cyclopropanol when it was intra-molecularly attacked by the α-carbon anion. Therefore, we tried to reduce the carbonyl group before the Henry reaction. Thus, treatment of compound **4** with triethylsilane and TFA gave compound **6** with high yield (98%). Compound **6**, which contained only one acidic site, could be easily converted to compound 7a by a double Henry type reaction by treatment with formaldehyde under basic condition [1]. To complete the synthesis of Fingolimod (**1**), the nitro group in compound 7a was reduced to amine, followed by a salt formation.

As described above, compound **6** [6,17] was the key intermediate in the synthesis of Fingolimod. We also tried an alternative way to prepared it. In this route, a much cheaper 3-bromopropionic acid was used instead of the relatively more expensive 3-nitropropionic acid. Thus, compound **3** was obtained by treatment of *n*-octylbenzene with 3-bromopropanoyl chloride under the presence of AlCl_3_. However, the direct displacement of the Br group to a NO_2_ group in compound **3** to form compound **4** using sodium nitrate and DMF failed, because the reason might be that the Br is a good leaving group, and the elimination of HBr to form an enone rather the nucleophilic substitution was preferred for compound **3**. To avoid the elimination reaction, a two-step operation was adopted to convert compound **3** to **6** by first reducing the carbonyl group by triethylsilane to form compound **5**, and then a S_N_2 displacement reaction to yield **6**. The yield of the S_N_2 displacement reaction was low under un-optimized conditions because some nitrite product was isolated.

In summary, a concise route for the synthesis of Fingolimod was reported from commercially available *n*-octylbenzene in **4** steps with an overall yield of 31%, and the atom economy for the whole route was 82.7%.

## Description of additional material

### 3-Bromo-1-(4-octylphenyl)propan-1-one (3)

To a flame-dried 500 mL 4-necked flask, was charged *n*-octylbenzene 24.39 g (0.128 mol), hexane 158 mL, and the reaction mixture was cooled to 5°C. To this mixture, a solution of 3-bromopropanyl chloride 25.0 g (0.146 mol) was added dropwise, followed by addition of AlCl_3_ 19.55 g (0.147 mol) in portion to control the reaction temperature under 10°C. The reaction mixture was allowed to stir at 0°C for 0.5 h, room temperature for 1 h, then reflux for 0.5 h. The reaction mixture was poured into a water-crushed-ice solution with vigorous stirring, the precipitate was collected and washed with water. The filtrate was extracted by ethyl acetate, and the combined organic phase was concentrated. The solid was combined and re-crystallized in petroleum ether to afford an off-white solid 31 g. yield:74.3%. M.p.:38–40°C. ^1^H NMR (400 MHz, CDCl_3_) δ 7.11 (m, 4H), 3.32 (t, *J* = 4.0 Hz, 2H), 3.13 (t, *J* = 4.0 Hz, 2H), 2.56 (t, *J* = 4.0 Hz, 2H), 1.59 (m, 2H), 1.23-1.35 (m, 10H), 0.87 (t, *J* = 6.8 Hz, 3H); ^13^C NMR (CDCl_3_) δ 198.8, 143.1, 132.6, 128.4, 128.1, 42.8, 36.0, 31.8, 31.3, 29.8, 29.4, 29.2, 26.5, 22.5, 14.3.

### 1-Bromo-3-(4-octylphenyl)propane (5)

To a flam-dried 250 mL 4-necked flask, was added 7.92 g (24.4 mmol) of compound **3** and 18.6 mL TFA. The reaction mixture was cooled to 10°C, and a solution of 5.65 g (48.7 mmol) of triethylsilane was added dropwise. The reaction mixture was allowed to stir at 10°C for 30 min, and then at room temperature for 4 h. The reaction mixture was poured into water with crushed-ice under vigorous stirring, and the pH was adjusted to 8 by NaHCO_3_. The mixture was extracted by petroleum ether 100 mL × 3, and the organic phase was combined, washed with brine, dried by Na_2_SO_4_, filtered, and concentrated to afford a yellow liquid, which was subjected to flash chromatography (EA:Hex = 1:8) to afford a colorless oil (7.46 g. Yield: 98.4%). ^1^H NMR (400 MHz, CDCl_3_) δ 7.12 (s, 4H), 3.18 (t, *J* = 8.0 Hz, 2H), 2.90 (t, *J* = 4.0 Hz, 2H), 2.57 (t, *J* = 4.0 Hz, 2H), 1.54-1.67 (m,4H), 1.24-1.34 (m, 10H), 0.88 (t, *J* = 6.8 Hz, 3H); ^13^C NMR (CDCl_3_) δ 136.1, 133.6, 128.4, 128.0, 36.1, 34.4, 33.4, 31.9, 31.3, 29.7, 29.4, 29.2, 28.8, 22.5, 14.1.

### Nitro-3-(4-octylphenyl)propane (6)

Method A: To a mixture of 9.59 g (30.8 mmol) of compound **5** and 44 mL of DMF, was added 8.47 g (122.8 mmol) of NaNO_2_ at 0°C. The reaction mixture was allowed to stir at 0°C for 0.5 h, then at room temperature for 6 h. The reaction mixture was poured into 200 mL of iced-water, extracted with petroleum ether 100 mL × 3. The organic layer was combined, washed with brine, dried by Na_2_SO_4_, filtered, and concentrated to afford a yellow liquid, which was purified by flash chromatography (EA:Hex = 1:8) to afford a colorless oil (3.00 g. Yield: 35.1%). Method B: By the same procedures as described in making compound **5** using compound **4** and triethylsilane as reactants. ^1^H NMR (400 MHz CDCl_3_) δ 7.07-7.13 (m, 4H), 4.36 (t, *J* = 7.2 Hz, 2H), 2.69 (t, *J* =7.5 Hz, 2H), 2.57 (t, *J* = 4.0 Hz, 2H), 2.31 (m, 2H), 1.55-1.61 (m, 2H), 1.24-1.34 (m, 10H), 0.88 (t, *J* = 7.0 Hz, 3H); ^13^C NMR (CDCl3) δ 139.8, 139.1, 128.4, 128.2, 89.5, 63.3, 36.1, 31.9, 31.3, 29.7, 29.4, 29.2, 28.83, 28.81, 22.5, 14.1.

### 3-Nitro-1-(4-octylphenyl)propan-1-one (4)

Compound 4 was prepared by the same method as described in the preparation of compound **3** using *n*-octylbenzene and 3-nitropropanoyl chloride (making from 3-nitropropanoic acid [18] and thionyl chloride) as starting material. Yield: 85%. ^1^H NMR (400 MHz CDCl_3_) δ 7.07-7.13 (m, 4H), 4.36 (t, *J* = 7.2 Hz, 2H), 2.69 (t, *J* =7.5 Hz, 2H), 2.57 (t, *J* = 4.0 Hz, 2H), 2.31 (m, 2H), 1.55-1.61 (m, 2H), 1.24-1.34 (m, 10H), 0.88 (t, *J* = 7.0 Hz, 3H); ^13^C NMR (CDCl_3_) δ 139.8, 139.1, 128.4, 128.2, 89.5, 63.3, 36.1, 31.9, 31.3, 29.7, 29.4, 29.2, 28.83, 28.81, 22.5, 14.1.

### 2-Nitro-2-(4-octylphenylethyl)propane-1,3-diol (7a)

To a four-necked round bottom of flask, was added 0.6 g (2.17 mmol) of compound **6** and 10 mL 1,4-dioxane, then 3.3 g of polyformaldehyde and 4 drops of TEA. The reaction was allowed to stir at 70°C for 4 h till all starting material was consumed. The solvent was removed under reduced pressure, and the residue was purified by flash chromatography (EA:Hex = 1:3) to afford the title compound as a white solid. Yield: 41.1%, M.P.: 99–101°C. ^1^H NMR( 400 MHz, CDCl_3_) δ: 7.09 (d, *J* = 8.0 Hz, 2H), 7.06 (d, *J* = 8.0 Hz, 2H), 4.24 (d, *J* = 12.0 Hz, 2H), 4.06 (d, *J* = 12.0 Hz, 2H), 2.53-2.57 (m, 4H), 2.16-2.20 (m, 2H), 1.54-1.59 (m, 2H), 1.24-1.34 (m, 10H), 0.84 (t, *J* = 6.0 Hz, 3H); ^13^C NMR (CDCl_3_) δ 136.1, 133.6, 128.4, 128.0, 75.1, 36.1, 33.4, 31.9, 31.3, 29.7, 29.4, 29.2, 28.8, 22.5, 14.1.

### 2-Amino-2-(4-octylphenylethyl)propane-1,3-diol HCl (1)

To a 100 mL reactor, was added 0.3 g (0.89 mmol) of compound **7a**, 10 mL of ethanol, and 30 mg of 10% Pd/C. The reaction mixture was allowed to stir at room temperature for 15 h under a 0.3 MPa hydrogenation condition. The reaction was filtered through a pad of *Celite*, and washed with ethanol. The combine filtrate was concentrated under reduced pressure to afford a white solid, which was treated with 20 mL of saturated HCl in ethanol, and the plate solid (0.28 g) was collected as the salt form of the title compound. Yield : 92%. M.p.: 106 ~ 108°C (lit.107-108°C). ^1^H NMR (400 MHz,CDCl_3_) δ: 7.08 (s, 4H, Ar-H), 3.62 (s, 2H), 3.54 (s, 2H), 2.52-2.60 (m, 8H), 1.69-1.70 (m, 2H), 1.57 (m, 2H), 1.20-1.25 (m, 10H), 0.87 (t, *J* = 6.0 Hz, 3H); ^13^C NMR (CDCl_3_) δ 139.8, 139.1, 128.3, 128.2, 61.1, 60.5, 34.9, 33.4, 31.4, 31.2, 29.0, 28.83, 28.81, 28.1, 22.2, 14.1.

## Abbreviations

TEA: Triethylamine
TFA: Trifluroacetic acid
DMF: *N*,*N*-Dimethylformamide
S_N_2: Nucleophilic Substitution
